# The Availability of Neutral Cyan, Green, Blue and Purple Colors from Simple D–A Type Polymers with Commercially Available Thiophene Derivatives as the Donor Units

**DOI:** 10.3390/polym9120656

**Published:** 2017-11-29

**Authors:** Lingqian Kong, Min Wang, Xiuping Ju, Jinsheng Zhao, Yan Zhang, Yu Xie

**Affiliations:** 1 Dongchang College, Liaocheng University, Liaocheng 252059, China; lingqiankong@126.com (L.K.); jxp1127@163.com (X.J.); 2 Department of Chemistry, Liaocheng University, Liaocheng 252059, China; zhang_yan1219@126.com; 3 Liaocheng People’s Hospital, Liaocheng 252000, China; wangmin1724@163.com; 4 College of Environment and Chemical Engineering, Nanchang Hangkong University, Nanchang 330063, China

**Keywords:** quinoxaline, thiophene derivatives, donor-acceptor polymers, electrochromic, spectroelectrochemistry

## Abstract

In this paper, the Stille coupling reaction was used to prepare four donor–acceptor–donor (D–A–D) type monomers. For this purpose, 2,3-bis(4-methoxyphenyl) quinoxaline was used as the acceptor unit, and thiophene derivatives (3,4-ethylenedioxythiophene, or EDOT; 3-methoxythiophene, or MOTh; 3-methylthiophene, or MTh; and thiophene, or Th) were used as the donor units. The monomers were polymerized to the corresponding polymers by the cyclic voltammetry (CV) or potentiostatic method. The band gaps and the adsorption profiles of the polymers were finely tuned with the incorporation of the different thiophene units. All four polymers have low band gaps, and switched between the colored neutral states and the highly transmissive oxidized state. We were successfully able to obtain the valuable neutral colors of cyan, green, blue, and violet for the polymers employing EDOT, MOTh, MTh, and Th as the donor unit, respectively. Furthermore, electrochromic kinetic investigations showed that all four polymers displayed excellent optical contrasts (Δ*T*%), fast switching times, high coloration efficiencies, and robust stabilities, indicating that these four polymers are probably promising choices for developing electrochromic devices.

## 1. Introduction

In recent years, conjugated polymers have been considered potential candidates for the active materials used in the preparation of many types of electrochromic devices, including energy-saving intelligent windows, none-emissive displays, and rearview mirrors for automobiles, as a result of their flexibility, fast color-changing speed, apparent color contrast, and tunable colors by structure modification [1,2,3]. Although a large variety of polymers have been reported, only a few of them possess the required color changes and switching properties for the construction of high performance electrochromic devices [4,5]. The structures of electrochromic conducting polymers (ECPs) can be synthetically modifiable, yielding a variety of anodically or cathodically coloring materials [6]. The accessibility of cathodically coloring ECPs with complete palettes of neutral state colors is a critical factor for the fabrication of high performance display devices, which could be boosted by developing synthetic methods to explore the structure–performance relationships of the polymers [7,8]. 

The primary colors are the basic colors that cannot be obtained by mixing other colors. In general, the superimposed primary colors (RGB) are red, green, and blue, while the subtractive primary colors (CMKY) are red, yellow, and cyan. Any color could be obtained by mixing the primary colors together [6]. The pursuit of ECPs with the primary colors has been the focus of research in the electrochromic fields [9,10]. According to the statistics within the published literature to date, most of the reported ECPs have presented red or blue colors in their neutral state. Therefore, those who have been trying to get neutral green polymers have a great interest in realizing the full color spectrum on the basis of the color-mixing theory, which is based on the RGB color spaces [11]. Besides, the neutral cyan polymers are also very limited, which restrict the fine tune of the colors over the entire visible regions on the base of the CMKY color space [8,12]. 

Among the approaches for getting this type of polymer, the donor–acceptor strategy appears to be the most useful method, which is characterized by constructing the polymer backbone by the alternate electron donor unit and the electron deficient unit [6]. The band gaps of donor–acceptor (D–A) type polymers could be fine-tuned through the reasonable combination of the donor unit and the acceptor unit, which can be implemented by two possible approaches. The first approach lies in the modification of the molecular structure of the donor unit or the alteration in the number of donor units incorporated in the polymer backbone [13,14]. Similarly, the second approach is characterized by the modification on the acceptor unit, as reported extensively. The donor units used usually consist of thiophene and its derivatives, including thiophene [3], diakoxythiophene (DalkOTs) [15], propylenedioxythiophene (PPETh) [13], thieno[3,2-b]thiophene [16], and benzodithiophene, etc. [17]. The selection of the donor unit and the number used determines to a great extent the color of the obtained polymers. Several electron-deficient units have been taken as the acceptor units, such as thien[3,4-b]pyrazine [18], benzothiadiazole [19], benzotriazole [20], and quinoxaline derivatives [21,22,23,24]. 

Some thiophene derivatives, including 3,4-propylenedioxythiophene (EDOT) are frequently used as the donor units for the preparation of D–A type polymers, which are often ideal ECPs with robust redox-cycling stabilities and apparent color changes [15,16,21]. The work of John R. Reynolds showed that DalkOTs could be used instead of propylenedioxythiophene (ProDOT) for the preparation of green-to-transmissive switching electrochromic polymers [16]. Sallenave et al. prepared a series of D–A type polymers by combining substituted PPETh and functionalized quinoxalines together, and the resultant polymers showed shades of blue to green as increasing the strength of the acceptor substituted on the diphenyl quinoxaline unit [14]. In an earlier study, Atilla Cihaner facilely obtained two CMKY colors, cyan and magenta, by using oxadiazole as the acceptor unit and thiophene or PPETh as the donor unit [8]. In Meng’s recent work, a neutral green polymer was obtained by incorporating a thieno[3,2,-b]thiophene derivative as the donor units, which illustrated the feasibility of using the unconventional thiophene derivative as the electron donor unit for the construction of high-performance ECPs [9]. In our recent works, some commercially available thiophene derivatives, including 3-methoxythiophene (MOTh), 3-methylthiophene (MTh), and 3,4-dimethoxythiophene, have been employed for the preparation of electrochromic polymers with desirable colors [21,22,23], which showed a convenient and inexpensive way for the exploration of high quality ECPs. However, such studies are very limited and should be strengthened. The electropolymerization method has been well documented for the preparation of a large quantity of polymer films with electrochromic properties. This method has the characteristics of handy operation and low costs, as well as extended polymer candidates for film fabricating. Since the electroplating process has realized large-scale production, as a kind of similar process, the electropolymerization method might be also used for mass production [10,11,12]. 

In this study, we report on the color dependence of some (D–A–D)*_n_* type neutral coloring polymers on the molecular structure of a thiophene derivative as the D unit, and 2,3-bis(4-methoxyphenyl) quinoxaline, a simple electron acceptor unit, as the “A” unit employed. A set of cathodically coloring polymers with neutral cyan, green, blue, and purple colors were facilely prepared as EDOT, MOTh, MTh, and thiophene (Th), respectively. These were taken as the donor units, while the acceptor unit was kept the same. The results of the present study considerably simplify the synthetic procedures for valuable neutral coloring systems, and thus promote the industrial magnification for electrochromic device applications. To the best our knowledge, there are very limited reports devoted to successive color tuning among primary colors by finely tuning the structure of commercially available thiophene derivatives. 

## 2. Experimental 

### 2.1. Materials

**Scheme 1:**
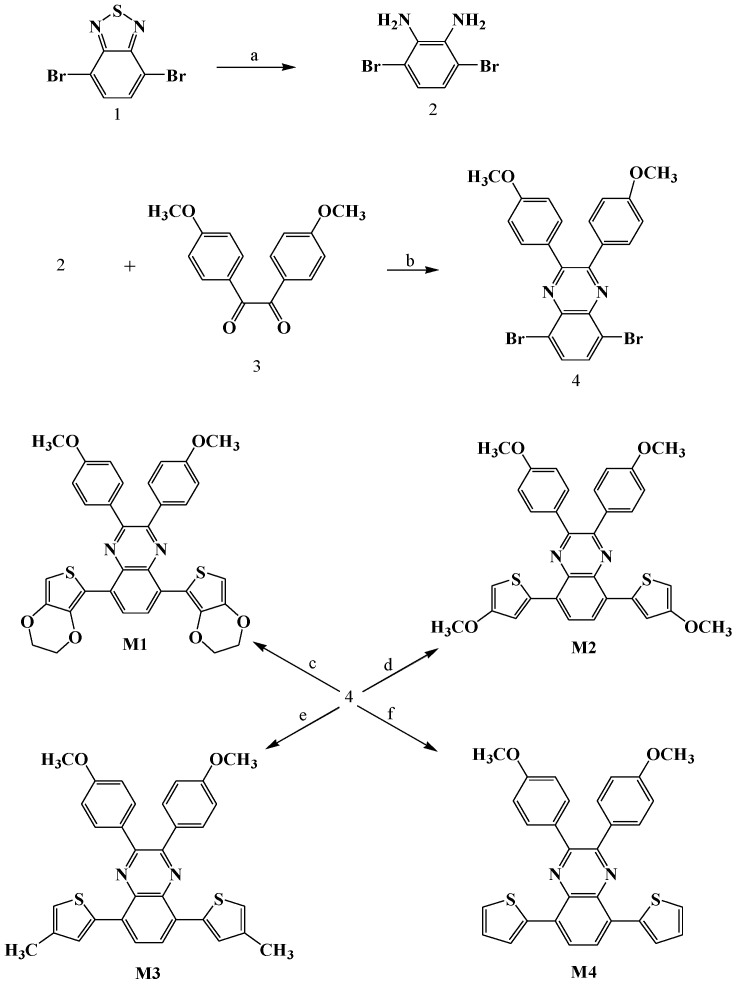
The synthetic route of the monomers and reaction mixtures, including: compound **4**, Pd(PPh_3_)_2_Cl_2_ (2% molar ratio of the organotin compounds), dry toluene, the organotin compounds of 3,4-ethylenedioxythiophene (EDOT) (**M1**), 3-methoxythiophene (MOTh) (**M2**), 3-methylthiophene (MTh) (**M3**), and thiophene (Th) (**M4**), refluxed under argon atmosphere for 24 h.

Thiophene and its derivatives are available from Alfa Aesar (Chemical Co., Ltd., Shanghai, China), and their corresponding 2-substitutued tributylstannane compounds were prepared with the procedures reported previously [21,25,26]. The acceptor 2,3-bis(4-methoxyphenyl) quinoxaline (compound **4** in Scheme 1) was also obtained by the condensation reaction between 3,6-dibromo-1,2-phenylenediamine and *p*-anisil, as described previously [21,25,26]. Other reagents used, including catalysts, solvents, and supporting electrolytes, were all purchased from Sigma-Aldrich Company Ltd. (Shanghai, China) through commercial channels. The formation of monomers in the synthesis process was monitored by thin layer chromatography using GF254 silica gel-backed plates, which was obtained from Sinopharm Chemical Reagent Beijing Co., Ltd. (Beijing, China), and observed under UV light. The monomers were purified with column chromatography using silica gel. 

### 2.2. Analysis Method 

^1^H NMR and ^13^C NMR spectra were recorded on a Varian AMX 400 spectrometer (Varian Inc., Santa Clara, CA, USA), and tetramethylsilane was used as the internal standard. A homemade single compartment cell was used for electrochemistry or spectroelectrochemical measurements with Pt wire (1 mm diameter) or indium-tin-oxide-coated (ITO) glass (Solems, 10 ohm sq^−1^, obtained from Zhuhai Kaivo Optoelectronic Technology Co., Ltd., (Zhuhai, China)) as the working electrode, and a platinum ring (1 mm diameter) and Ag wire (calibrated vs. ferrocene) as the counter electrode and the pseudo-reference electrodes, respectively. A step profiler (D-100, KLA-Tencor, obtained from KLA-Tencor Co., Ltd., Milpitas, CA, USA) was used for the evaluation of the thickness of the polymer film. The images for the surface microstructure were visualized by a thermionic field emission SEM (Hitachi SU-70, obtained from Hitachi, Ltd., Tokyo, Japan). UV–Vis–Near infrared spectra (NIR) were recorded on a spectrophotometer (Varian Cary 5000, obtained from Varian Inc., Santa Clara, CA, USA), and chromaticity measurements were also obtained with the spectrophotometer with the aid of the Chroma software. For this purpose, Illuminant C and a 2° observer were used. The electronic levels and the configuration optimization were conducted using density function theory (DFT) with the B31YP/lan12dz functional. The Gaussian 09 program was employed for the above optimization process. 

### 2.3. Synthesis

Scheme 1 gave the synthetic procedures of the monomers involved in the present study. The synthetic route for all of the thiophene organotin compounds and compound **4** have been described in detail in previous reports, and can be obtained by the reported method (see Supplementary Materials Figure S1) [21,25,26]. The synthetic route for the four monomers **M1**–**M4** followed a classical Stille coupling reaction. A closed device with inert gas holding virtue was used for the performance of the synthesis, which consisted of a round-bottom flask, a condenser connected with a three-way valve, and a balloon connected with a three-way valve. In the round-bottom flask, 20 mmol of the tributyl stannane compounds of thiophene or its derivatives, and 4 mmol of compound **4** were dissolved in 60 mL of toluene. The mixture solution was stirred under argon atmosphere, and 0.5 mmol of Pd(PPh_3_)_2_Cl_2_ was added to the mixture. Then, the air in the reactor was replaced by argon, and the inert atmosphere of the reactor was maintained by the argon in the balloon. The reaction mixture was refluxed for 24 h. After cooling down, toluene was removed through vacuum evaporation, and the corresponding monomers were purified from the above crude products by column chromatography, and the silica gel was used as the stationary phase. 

Monomer **M1**. *n*-hexane/CH_2_Cl_2_ (3:1) as the eluent. **M1** was obtained as an orange red solid with a yield of 67%. ^1^H NMR (CDCl_3_, 400 MHz, ppm): δ = 8.59 (s, 2H, ArH), 7.73 (d, *J* = 8.79, 4H, ArH), 6.91 (d, *J* = 8.8, 4H, ArH), 6.55 (s, 2H), 4.40 (m, 8H), 3.86(s, 6H). ^13^C NMR (CDCl_3_, 101 MHz, ppm): δ = 160.10, 150.18, 141.28, 140.15, 136.81, 131.91, 131.24, 128.40, 127.59, 113.57, 113.38, 102.91, 64.89, 64.30, 55.23 (Figure S2). 

Monomer **M2**. *n*-hexane/CH_2_Cl_2_ (4:1) as the eluent. **M2** was obtained as an orange red solid with a yield of 64.5%. ^1^H NMR (CDCl_3_, 400 MHz, ppm): δ = 8.03 (s, 2H, ArH), 7.74 (d, *J* = 8.0, 4H, ArH), 7.53 (s, 2H), 6.94 (d, *J* = 8.6, 4H, ArH), 6.48 (s, 6H), 3.88 (s, 6H), 3.89 (s, 6H). ^13^C NMR (CDCl_3_, 101 MHz, ppm) δ = 160.31, 158.03, 151.17, 137.62, 137.09, 131.91, 131.22, 130.47, 126.16, 117.97, 113.67, 100.74, 57.23, 55.31 (Figure S3). 

Monomer **M3**. *n*-hexane/CH_2_Cl_2_ (5:1) as eluent. Orange solid (yield, 70%). ^1^H NMR (CDCl_3_, 400 MHz, ppm): δ = 8.06 (s, 2H, ArH), 7.75 (d, *J* = 8.64, 4H, ArH), 7.67 (s, 2H), 7.11 (s, 2H),6.93 (d, *J* = 8.8, 4H, ArH), 3.98 (s, 6H), 2.38 (s, 6H). ^13^C NMR (CDCl_3_, 101 MHz, ppm) δ = 160.27, 150.98, 138.61, 136.98, 131.90, 131.37, 130.85, 128.52, 126.35, 124.57, 113.64, 55.30, 15.83 (Figure S4). 

Monomer **M4**. *n*-hexane/CH_2_Cl_2_ (5:1) as eluent. Bright yellow solid (yield, 77.3%). ^1^H NMR (CDCl_3_, 400 MHz, ppm): δ = 8.11 (s, 2H, ArH), 7.85 (dd, *J* = 3.56, 2H), 7.74 (d, *J* = 8.64, 4H, ArH), 7.52 (dd, *J* = 4.96, 2H), 7.18 (t, *J* = 3.68, 2H), 6.92 (d, *J* = 8.8, 4H, ArH), 3.85 (s, 6H). ^13^C NMR (CDCl_3_, 101 MHz, ppm) δ = 160.32, 151.17, 138.85, 136.93, 131.88, 131.33, 130.98, 128.75, 126.95, 126.52, 126.18, 113.70, 55.30 (Figure S5). 

## 3. Results and Discussion

### 3.1. Electrochemical Polymerization

**Figure 1:**
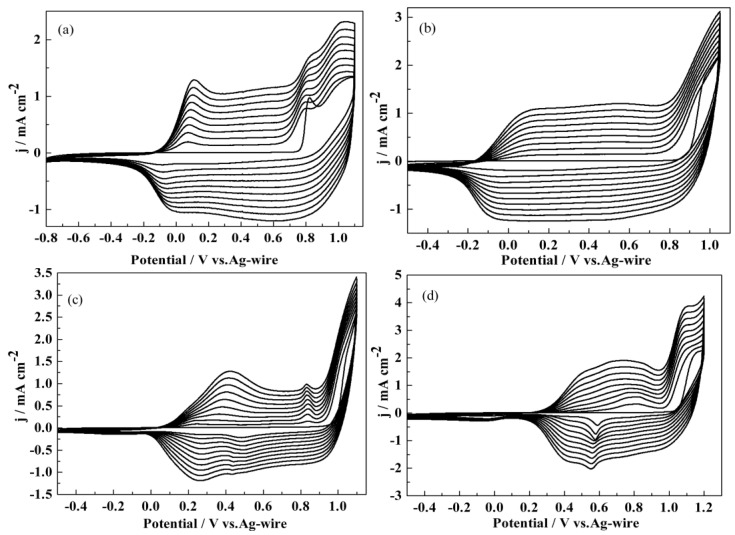
Cyclic voltammograms of **M1** (**a**), **M2** (**b**), **M3** (**c**), and **M4** (**d**) in acetonitrile/dichloromethane (1:1) solution containing 0.2 M TBAPF_6_ at a scan rate of 100 mV s^−1^. The potential of Ag wire was calibrated to be 0.03 V vs. SCE using the E_1/2_ of the ferrocene calibration method.

Cyclic voltammetry (CV) was used to study the polymerization ability and redox properties of the monomers, and the electrolyte used was 0.1 M tetra-*n*-butylammonium hexafluorophosphate (TBAPF_6_) dissolved in acetonitrile/dichloromethane (1:1, by volume). The electrolyte is used in the present study for all of the electrochemical measurements without specific remainders. The respective monomer was dissolved in the electrolyte with a concentration of 5 mM, and then the CV measurement was conducted with a sand rate of 100 mV/s, until 10 cycles of the CV were completed. The scanning potential windows of the monomers and their graphic shape are shown in Figure 1. As the number of scanning cycles increased, the redox currents of the monomers increased continuously, and the surface of the working electrodes were covered with materials with different colors, which is an indication of the occurrence of the polymerization [20]. From the first CV cycle of the monomers, the anodic oxidation of the monomers was observed, and the resultant charge carrier species were considered the precursors for the polymerization reactions. The onset oxidation potentials (*E_onset_*), which are listed in Table 1, moved slightly more negative following the increasing electron-donating abilities of the thiophene derivatives, and in the order of Th < MT < MOT < EDOT. Table 1 also listed the highest occupied molecular orbital (HOMO) levels of four monomers from the geometry optimizations on the basis of the theoretical calculations, which clearly showed that the HOMO energy levels of the monomers followed the order of **M1** > **M2** = **M3** > **M4**. For the D–A–D type monomers, the HOMO levels could be considered mainly concentrated on the two capping donor units; i.e., the HOMO levels of the monomers were substantially determined by the thiophene derivatives [21]. The decreasing HOMO levels of the monomers from **M1** to **M4** were attributable to the decreasing electron-donating abilities of the thiophene derivatives from EDOT to thiophene, correspondingly. Based on the above discussions, the order of the *E_onset_* values was consistent with the magnitude order of the HOMO energy levels, which indicated that the data from the experiment was in agreement with the data from the theoretical calculations. The high HOMO levels are beneficial to the formation and stabilization of the cationic radicals resulting from the oxidation of the monomers, as well as the improvement of the polymerization abilities of the monomers [25]. 

### 3.2. Redox Behaviour

**Figure 2:**
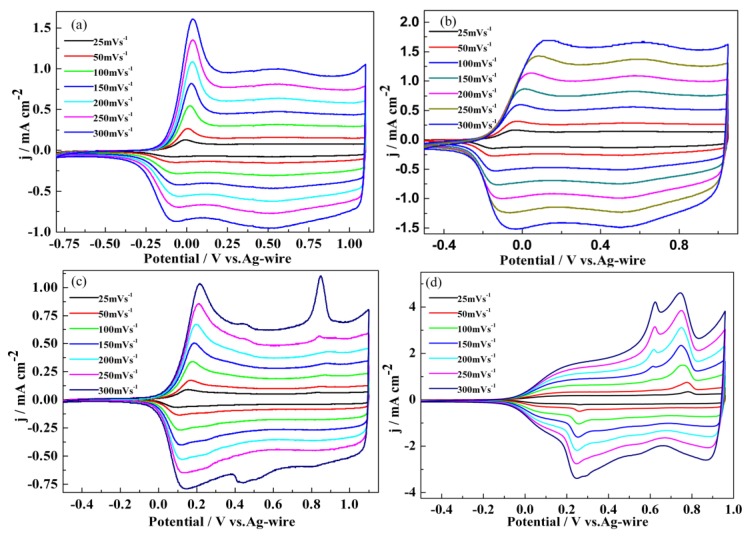
Cyclic voltammetry (CV) curves of the **P1** (**a**), **P2** (**b**), **P3** (**c**), and **P4** (**d**) films at different scan rates from 300 mV s^−1^ to 25 mV s^−1^ in the monomer-free 0.2 M TBAPF_6_-ACN-DCM solution.

The redox performance of the polymers was conducted from thin films coated on the Pt wire (three CV cycles), and the testing medium selected was the monomer-free electrolyte mentioned above. As for the conjugated polymer, the redox behavior originated from the doping/dedoping process driven by the applied voltage on it, and the reversibility of the redox process substantially lay on the stabilization of the charge carriers by the counter anion. Except for the polymer of **P4**, the redox curves of the remaining three polymers have similar shapes, i.e., broad, flat, and rectangular (Figure 2). In contrast, the redox curves of polymer **P4** (Figure 2d) was somewhat slim, with the appearance of the apparent redox peaks located at 0.7 V/0.5 V. The widths of the rectangular peaks were 1.19 V, 0.88 V and 0.66 V, respectively, with **P1** having the widest peak, followed by **P2** and **P3** (Figure 2). The rectangular redox peak of the polymers indicated that the polymers have a high degree of conjugation [21]. In this case, the magnitude of the conjugation length is in the order of **P1** > **P2** > **P3** > **P4**, which is consistent with the strength of the electron-donating abilities of the donor units along the backbone of the polymers [25]. In addition, the *E_onset_* of the polymers were also listed in Table 1. The *E_onset_* of **P4** was 0.17 V, which is far higher than that of **P1**, **P2**, and **P3**, owing to the relatively weak electron-donating ability of the thiophene ring, compared with the other three donor units of **P1**, **P2,** and **P3** (Figure 2). Out of expectation, the onset oxidation potential of **P2** (*E_onset_* = −0.17 V) was lower than that of **P1** (*E_onset_* = −0.11 V). This might be because the electron-donating effect of EDOT, as the donor of **P1,** cannot offset its sterically hindered effect, which is caused by the side chain of the 3,4-dioxane ethylene unit. In addition, there are some slight differences in the *E_onset_* values between the polymers possessing the same donor unit and the different acceptor units, which indicated that the acceptor unit also contributes to the HOMO energy levels of the polymer to a limited extent [26]. 

**Figure 3:**
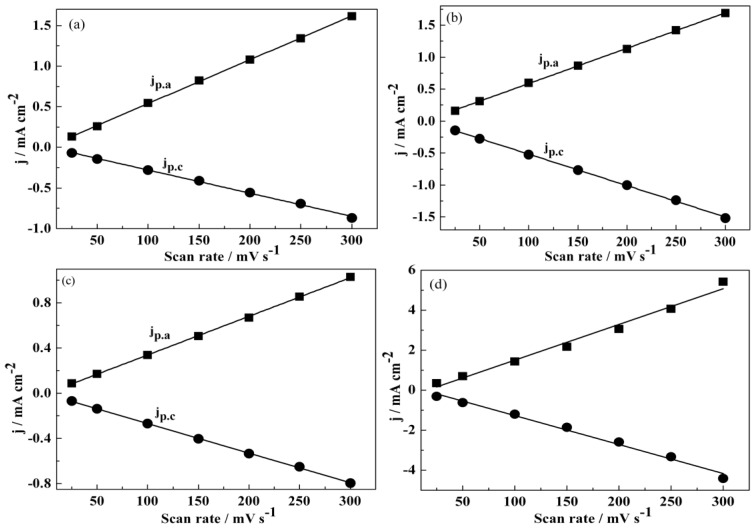
Scan rate dependence of the anodic and cathodic peak current densities graph of the doping/dedoping process for the **P1** (**a**), **P2** (**b**), **P3** (**c**) and **P4** (**d**) films.

Figure 3 presented the relationships between the peak currents and the scanning speed, which rendered the direct evidence for confirmation of the reversibility of the doping/dedoping process of the polymers. Obviously, the perfect linear relationship was present between two parameters of current densities and scan rates, which indicated that the presence of the reversibility of the doping/dedoping process and the good conductivity of four polymers. The linear relationships between the scan rates and the peak current densities of the polymers also indicated that the conductive polymers prepared by electrochemical deposition are tightly bonded to the electrodes, and the charge transfer between the polymer films and the electrode are smooth [28]. 

In order to study the electrochemical stabilities of the polymers, the CV of four polymer films were conducted in the repeated patterns for more than 1000 cycles. The CV curves of the first, 500th, and 1000th cycles for four polymer films were shown in Figure S6. After 1000 cycles, the total charge loss of the **P1**, **P2**, **P3,** and **P4** films were 3.50%, 4.21%, 4.25%, and 6.17%, respectively. The loss during the first 500 cycles was more than 75% of the total loss for the four polymers. From the above data, it was inferred that the four polymer films had wonderful stabilities, which gives them important application prospects in the construction of commercial devices. In addition, it is easy to find that **P1** has the minimum loss of total charge between the 1–1000th cycles, which is mainly related to the increased stability of the generated polaron/bipolarons of **P1** compared with that of the other polymers [18]. It was interesting to find that the stabilities of the polymers have positive correlations with the contents of polar oxygen atoms in their polymer chains. 

### 3.3. Morphology 

**Figure 4:**
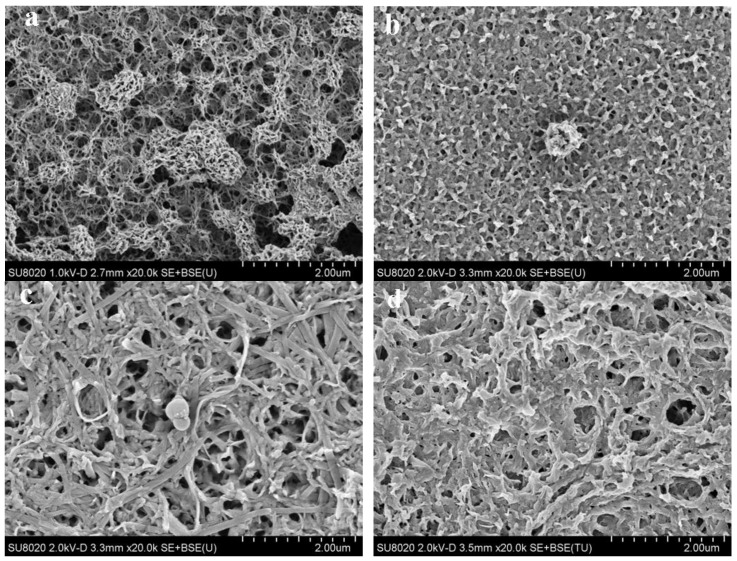
SEM images of **P1** (**a**), **P2** (**b**), **P3** (**c**), and **P4** (**d**) films deposited potentiostatically onto ITO electrodes.

**Figure 5:**
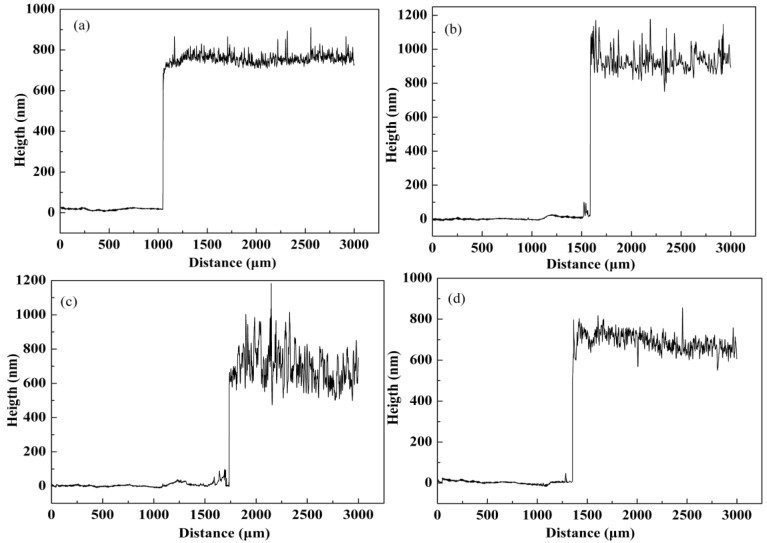
The step profiler images of **P1** (**a**), **P2** (**b**), **P3** (**c**), and **P4** (**d**) films deposited potentiostatically onto ITO electrodes.

The bulk morphologies and microstructures of the polymers were studied using scanning electron micrographs (SEM). The polymer films were prepared by the constant potential method on ITO electrodes and dedoped before characterization. The SEM images of **P1**, **P2**, **P3,** and **P4** are shown in Figure 4. As seen in Figure 4a, the **P1** film shows an irregular mesh structure, and there are some holes between the crossover networks. The **P2** film (Figure 4b) exhibits a homogeneous and flat surface and is decorated with small sheet-shape objects and small cavities. The **P3** film consists of disordered line segments, which are randomly stacked in a flat structure, and are accompanied by the resulting cavity structure. The **P4** film (Figure 4d) presents a laminated structure, and consists of relatively lager sheet-shape objects, and some holes are also dispersed on the film. The porous structure of the polymer films promotes the doping/dedoping process of the counter anions within the polymer films during the redox cycling, which has a marked impact on the electrical and optical performance of the polymers. Estimations of the thickness and roughness of the polymer films were carried out by using the step profiler. The thicknesses of the **P1**, **P2**, **P3,** and **P4** films were measured as 772 nm, 942 nm, 740 nm, and 720 nm, respectively. As shown in Figure 5, the rugged scanning curves demonstrate that the surface of the polymer film is uneven, which is consistent with the porous structure obtained by SEM measurements [26]. 

### 3.4. Optical Properties of the Monomers and Films 

**Figure 6:**
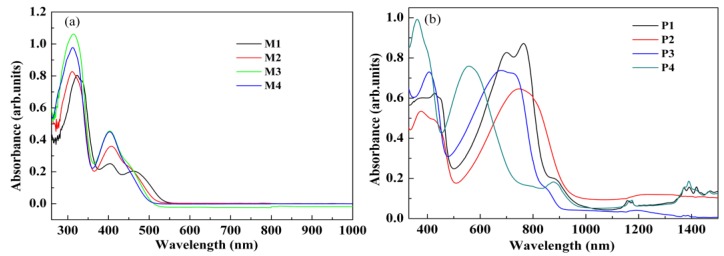
Optical absorption profiles of the monomers in dichloromethane (DCM) (**a**), and their respective polymers deposited on the ITO electrode in the dedoped state (**b**).

It can be clearly seen from Figure 6 that there were two evident absorption bands in the spectrum of **M1**; one was located at 322 nm, and the other at 461 nm. Similarly, the other three monomers also exhibited two absorption bands at 310 nm and 407 nm for **M2**, 314 nm and 403 nm for **M3,** and 312 nm and 403 nm for **M4**. The double peak pattern is a typical feature of the D–A–D compounds, which consists of a high-energy absorption band and a low-energy absorption band. The former band originates from the donor unit, and the latter band originates from the charge transfer between the donor unit and the acceptor unit [10]. From **M1** to **M4**, there were apparent bathochromic shifts of the low energy absorptions due to the increasing electron-donating abilities of the D units, which enhanced the electron transfer between the two types of units on the premise of the unchanged acceptor unit [21,25]. Furthermore, the band gaps (*E_g_*) of the monomers were obtained from the formula usually used (*E_g_* = 1241/*E_onset_*). So, the *E_g_* values of **M1**, **M2**, **M3,** and **M4** were obtained as 2.32 eV, 2.39 eV, 2.42 eV, and 2.49 eV, according to onset absorption edges (λ*_onset_* = 534 nm for **M1**, 517 nm for **M2**, 511 nm for **M3,** and 499 nm for **M4**), respectively. The *E_g_* theoretical values of the four monomers were also obtained from the theoretical calculation based on the Gaussian 05 programs (Table 1). Obviously, these theoretical values were higher than the values of the optical *E_g_*, which might be the result of multiple factors, such as the solvent effect and the changes aroused from the transition from the solid to the gaseous state. 

**Figure 7:**
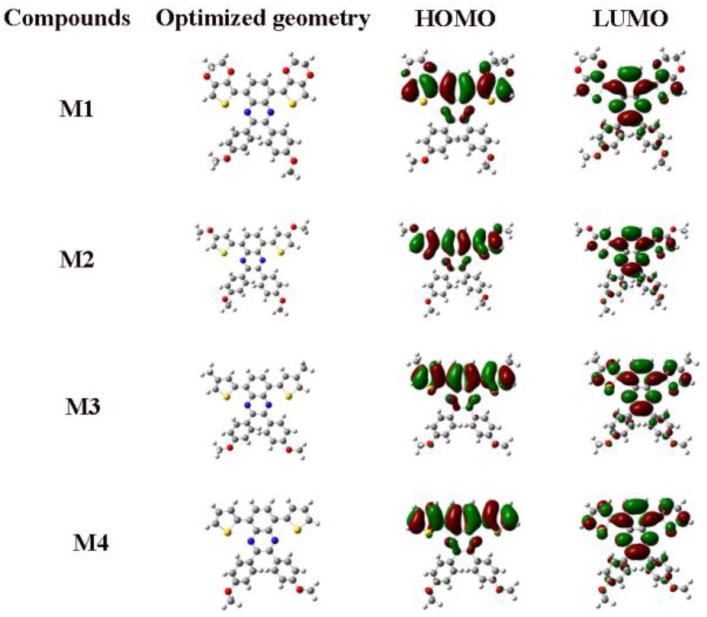
The optimized geometries and the molecular orbital surfaces of the HOMOs and LUMOs for the monomers.

For all of this, the relative order of the theoretical optical band gaps is consistent with that of the experimental values. Density functional theory was used to further research on the structures of the monomers, including the band gap, planar structure and HOMO–LUMO levels (Table 1 and Figure 7). As shown in Figure 7, the HOMO and LUMO orbital energies of the monomers were largely delocalized on the aromatic rings, which implied that a planar conjugated system was obtained. According to the difference in the distribution of the electron clouds in the HOMO and LUMO orbital energies, the phenomenon of charge transfer could be easily identified in the four D–A–D type monomers. 

The optical absorption profiles of the polymers in the neutral state were presented in Figure 6b. The pattern of double absorption bands is also applicable to the resultant polymers from the monomers, with the bands located at 427 nm and 764 nm for **P1**, 377 nm and 749 nm for **P2**, 406 nm and 679 nm for **P3**, and 362 nm and 560 nm for **P4**; the origination of the absorption bands were similar with that of the monomers [21,25]. Compared with the corresponding monomers, the maximum absorption peaks of the four neutral state films (**P1**, **P2**, **P3** and **P4**) experienced a red shift, which may be attributable to the extended conjugation length derived from polymerization. As shown in Table 1, the *E_g_* values of **P1**, **P2**, **P3,** and **P4** were calculated to be 1.48 eV, 1.36 eV, 1.49 eV, and 1.54 eV based on the formula of the optical band gap, respectively. Apparently, **P4** had the highest *E_g_* among all four polymers, which might be attributed to the weakest electron-donating abilities of Th as the donor unit. Unexpectedly, **P2** had a lower optical band gap than **P1**, although EDOT had a stronger electron-donating ability than MOTh The side chains of 3,4-dioxane ethylene on the EDOT unit had a higher steric hindrance effect than that of the methoxyl group on the MOBT unit, which resulted in the blue shift of the absorption spectrum of **P1** respective to that of **P2**. MTh has a weaker electron-donating ability than that of EDOT or MTh; as a result, **P3** has slightly larger *E_g_* values than **P1** and **P2**. Besides, the *E_g_* values of the polymers reported in the present study are slightly different than those of the same type of D–A type polymers with quinoxaline as the acceptor unit, which suggested that the colors of the polymers could be finely tuned through the modification of the structure of the quinoxaline derivatives [21,25]. In other words, the rational combination of a strong electron-donating group and a strong electron-accepting group is beneficial for decreasing the band gaps of the resultant polymers, as well as accessing the desired colors for high-quality display devices. The pseudo Ag wire reference electrode was calibrated in acetonitrile(ACN)–dichloromethane(DCM) (1:1, by volume) solution containing 0.2 M TBAPF_6_ and 0.005 M solution of ferrocene (Fc/Fc+).

### 3.5. The Dependence of Optical Absorption on the Biased Potentials

**Figure 8:**
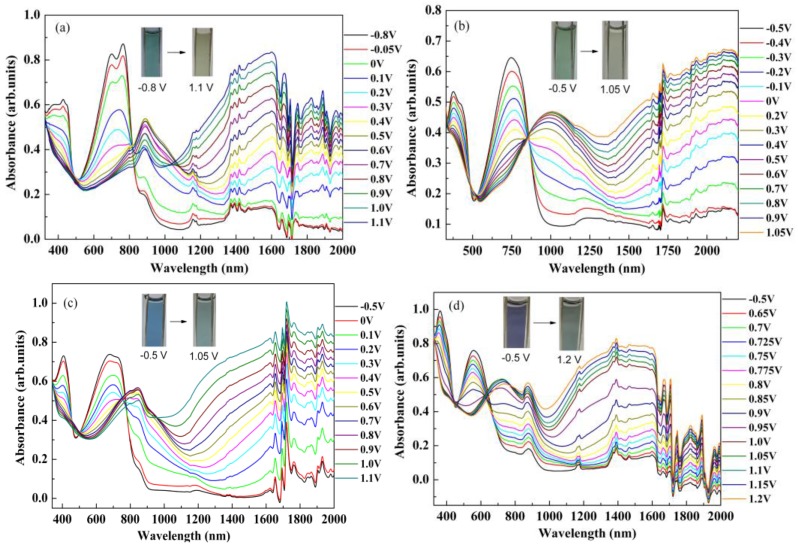
The variance in the optical absorption spectra of the polymer films on ITO electrodes in the monomer-free electrolyte when the biased potential was adjusted between −0.8 V and 1.1 V for **P1** (**a**); −0.5 V and 1.05 V for **P2** (**b**); −0.5 V and 1.1 V for **P3** (**c**); and −0.5 V and 1.2 V for **P4** (**d**).

The spectroelectrochemistry of the four polymer films was carried out to get detailed information about the changes in the conjugation structures of the polymers during the doping process, which is shown in Figure 8. A total charge of 2.5 × 10^−2^ C was deposited on the ITO electrode with an area of 0.8 × 1.8 cm, with the polymerization potential of 1.1 V for **P1**, 1.05 V for **P2**, 1.1 V for **P3**, and 1.2 V for **P4**. The polymers were switched to their neutral states before the beginning of the experiments. The changes in absorption profile were recorded in the electrolyte when the biased potential was switched from the negative potential to the positive potential in a gradually increased mode. The color change from the neutral to the oxidized states for each polymer is also presented in the inset of Figure 8. 

As expected, all four polymer films showed a dual-band absorption spectra in the initial negative potentials, which was a feature for the doctor-acceptor type polymer. The bands were centered at 427 nm and 764 nm for **P1**, 377 nm and 749 nm for **P2**, 406 nm and 679 nm for **P3**, and 362 nm and 560 nm for **P4**. The high-energy transition peaks (in the UV region) originated from the transition between the thiophene-based valence band and its anti-bonding counterpart, and the low-energy transition peaks (in the visible region) were due to the charge transfer between the donor unit and the acceptor unit. As shown in Figure 8a, for polymer **P1**, the intensities of the two types of bands were comparable to each other, which demonstrated that there was a perfect match between EDOT (the donor unit) and the acceptor unit, i.e., a smooth charge transfer occurred between them. Similarly, the phenomenon was also observed for the other three polymers (**P2**, **P3,** and **P4**), as shown in Figure 8b–d. For the absorption profiles, isosbestic points were displayed at around 817 nm for **P1**, 854 nm for **P2**, 765 nm for **P3**, and 634 nm for **P4**, which indicated that the four polymers were being interconverted between the neutral form and the radical cation. In addition, with the increase of applied voltage, the polymer films were gradually oxidized and the intensity of both bands decreased. A new band in the NIR region simultaneously occurred, which resulted from the formation of polarons and bipolarons. Color changes also accompanied the oxidation process; all of the polymers switched between the colored neutral states and the highly transmissive state. The colors of the four polymer films were assessed by using the CIE 1976 L*a*b* Color Space with a D65 illuminant, as shown in Table 2. The CIE 1931 XYZ chromaticity coordinates were also provided in Table 2. Here, a*b* values relate to the hue or saturation of a color (−a* and +a* are equal to green and red, −b* and +b* are equal to blue and yellow), and the value of L* represent the lightness of a color from 0 to 100 (black to white).

**Figure 9:**
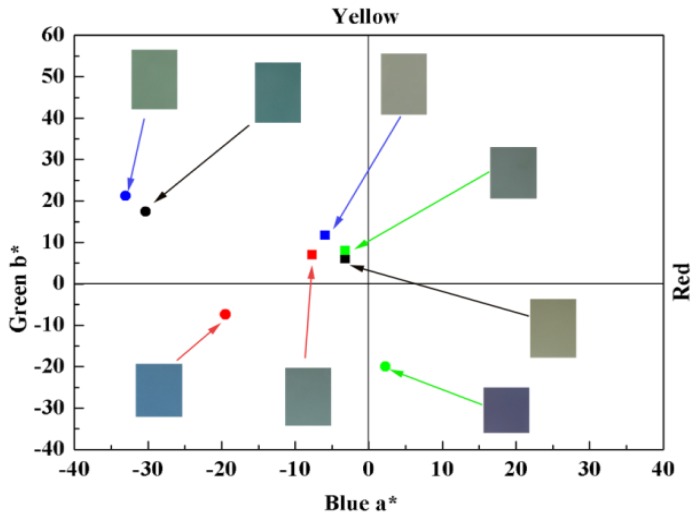
Colorimetry a*b* color coordinates of all the polymers in the different states. Circles refer to the reduced state, and squares refer to the oxidized state.

Figure 9 plotted the a*b* values of the polymers in different states. For the **P1** film in the neutral state, the data are −30.32 and 17.42, which suggested that **P1** presented a cyan color. With the polymer oxidized, the values of a*b* decreased, and the **P1** film expressed a transmissive tawny color (a* = −3.17, b* = 5.97) at the oxidized state. Meanwhile, the L* value increased from the neutral state to the oxidized state, which indicated an increasing lightness of the **P1** film. Similarly, the a*b* values of the **P2** film in the neutral state (a* = −33.03, b* = 21.24) indicated a green color, and the data (a* = −5.88, b* = 11.69) at the full oxidized state indicated a transmissive gray color. For the **P3** film, the data (a* = −19.46, b* = −7.40) in a neutral state indicated a blue color, which turned to a transmissive green (a* = −7.69, b* = 6.92) color in the doped state. At a biased potential of −0.5 V, the **P4** film exhibited a purple color (a* = 2.26, b* = −20.01), and the highly transmissive pale bluish green color (a* = −3.17, b* = 7.96) color at the oxidized state of the **P4** film. A consistent trend is that the L* values of all of the polymers increased, which demonstrated the increases of the perceived lightness of the films (see Table 2). 

### 3.6. Optical Switching Performance

**Figure 10:**
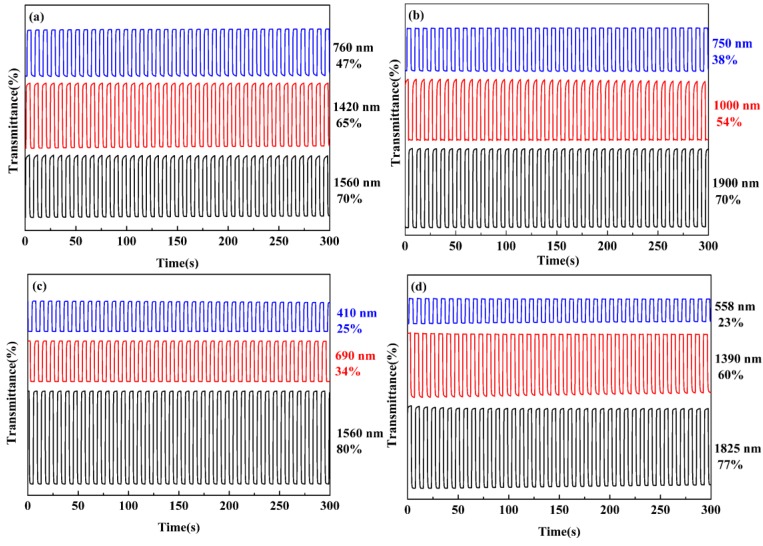
Transmittance changes of the four polymers by continuous monitoring at the indicated wavelength during the electrochromic switching in the electrolyte. (**a**) **P1**, −0.8 to 1.1 V; (**b**) **P2**, −0.5 to 1.15 V; (**c**) **P3**, −0.5 to 1.1 V; (**d**) **P4**, −0.5 to 1.2 V.

**Figure 11:**
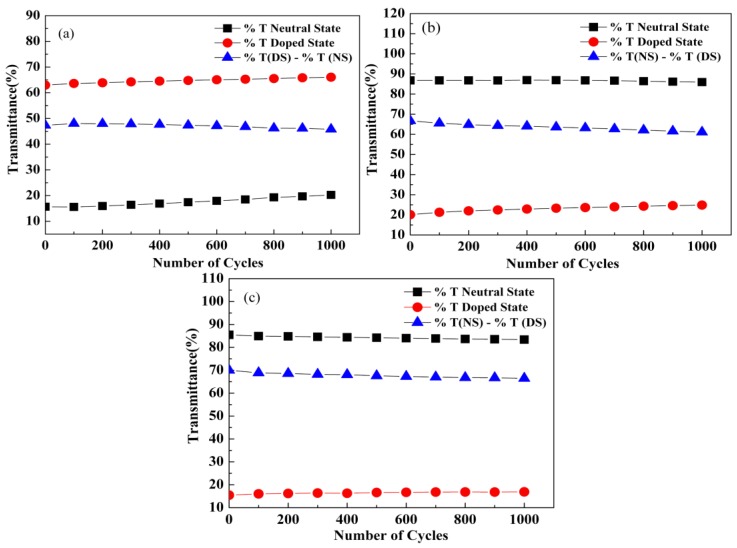
Electrochromic stabilities of **P1** at 760 nm (**a**), 1420 nm (**b**), and 1560 nm; (**c**) switched 1000 cycles in the monomer-free 0.2 M TBAPF6–ACN–DCM solution.

The optical switching performances of the four polymer films were explored by the double potential step chronoamperometry method, with a residence time of 4 s in the electrolyte. The percent transmittances as a function of time of the four polymers at different definite wavelengths were observed in Figure 10. The optical contrast (Δ*T*%) refers to a percent transmittance contrast during the doping/dedoping process, which is an important factor for appraising electrochromic materials. **P1** revealed 47% of the Δ*T*% value at the 760 nm within a full redox switch, and the data were 65% and 70% at the wavelength of 1420 nm and 1560 nm in the NIR region, respectively (Figure 10a). Figure 11 showed the electrochromic stability of the **P1** film at different given wavelengths, which is the change of optical contrast (Δ*T*%) after 1000 cycles of switching. **P1** displayed a loss of optical contrast was 1.5% (from 47.3% to 45.8%) at 760 nm, 4.6% (from 65.7% to 61.1%) at 1420 nm, and 3.6% (from 70.1% to 66.6%) at 1560 nm over 1000 switching cycles. The Δ*T*% values for **P2** were 38% at 750 nm, 54% at 1000 nm, and 70% at 1900 nm within the full redox switch (Figure 10b). After 1000 cycles of switching, the loss in Δ*T*% values for **P2** were 0.4% (from 38.0% to 37.4%) at 750 nm, 3.3% (from 54.0% to 50.7%) at 1000 nm, and 1.9% (from 70.1% to 68.2%) at 1900 nm, as indicated in Figure S7a–c.

As presented in Figure 10c, **P3** showed a Δ*T*% value of 25% at 410 nm, and the data were 34% and 80% at 690 nm and 1560 nm, respectively. Stability tests showed that the loss in Δ*T*% values for **P3** were 1.3% (from 25.4% to 1.3%) at 410 nm, 0.9% (from 34.0% to 33.1%) at 690 nm, and 0.5% (from 79.8% to 79.3%) at 1560 nm (Figure S8a–c). The optical contrasts for **P4** were found to be 23% at 558 nm, 60% at 1390 nm, and 77% at 1825 nm (Figure 10d). Similarly, the loss in Δ*T*% value for **P4** was 0.6% (from 23.6% to 23.0%) at 558 nm, 0.8% (from 60.6% to 59.8%) at 1390 nm, and 0.4% (from 77.1% to 76.7%) at 1825 nm (Figure S9a–c). Obviously, there was only a small loss for all of the polymers up to 1000 repeated switches, which demonstrated that the four polymers presented robust kinetic stabilities. All of the polymers reported in this study revealed relatively high optical contrasts in contrast with the similar structures of the D–A type polymers reported previously, such as poly(2,3-di(5-methylfuran-2-yl)-5,8-bis(2-thienyl)quinoxaline) (PMFTQ) and poly(2,3-di(5-methylfuran-2-yl)-5,8-bis(2-(3,4-ethylenedioxythiophene))quinoxaline) (PMFEQ) [25]. In addition, all four polymers had relatively high optical contrast values in the NIR region, which endowed the polymers with the indispensable merit for their utilization as the active materials for the NIR electrochromic device.

Response time (τ) is also known as the time required to complete 95% of the maximum Δ*T*% value when switched between the reduced state and the oxidized state. **P1** had excellent response times of 0.24 s at 760 nm, 1.21 s at 1420 nm, and 0.60 s at 1560 nm. For **P2**, the switching times were calculated as 1.00 s at 750 nm, 0.80 s at 1000 nm, and 1.80 s at 1900 nm, respectively. **P3** had relatively slow response times of 2.32 s at 410 nm, 3.00 s at 690 nm, and 0.83 s at 1560 nm. **P4** had switching times of 0.34 s at 558 nm, 0.83 s at 1390 nm, and 0.84 s at 1825 nm, which are comparable to the values of **P1**. It was clearly found that all of the response times for the four polymers were less than 2 s, except for the response times of **P3** at 410 nm and 690 nm. Among them, the **P1** and **P4** films exhibited faster response times in comparison with PMFEQ and PMFTQ, respectively [27]. The fast switching times of the four polymers indicated that the diffusion of these counter ions across the polymer films is relatively easy. 

The coloration efficiency (CE) is related to the charge utilization efficiency during the color-changing process, and the higher the CE values are, the more efficient the energy conservation [21,25]. The following equations are used for getting the CE values (*η*) [21,25].

$$\Delta \mathrm{OD}=\mathrm{lg}(\frac{T_{b}}{T_{c}})$$

$$\eta =\frac{\Delta \mathrm{OD}}{\Delta Q}$$

where *T*_b_ and *T*_c_ are the transmittances in the bleached and colored states at the given wavelength, respectively. ΔOD is the change in optical density at the given wavelength. ΔQ is the amount of ingress/egress charge per unit electrode area. *η* denotes the value of CE. The CE values of **P1** were calculated to be 264.7 cm^2^ C^−1^ at 760 nm, 247.6 cm^2^ C^−1^ at 1420 nm, and 300.0 cm^2^ C^−1^ at 1560 nm. For **P2**, the CE values were calculated as 185.4 cm^2^ C^−1^ at 750 nm, 135.3 cm^2^ C^−1^ at 1000 nm, and 254.4 cm^2^ C^−1^ at 1900 nm by the same method. For **P3**, the CE values measured using the above mentioned equations were 171.3 cm^2^ C^−1^ at 410 nm, 252.8 cm^2^ C^−1^ at 690 nm, and 350.0 cm^2^ C^−1^ at 1560 nm. As for **P4**, the data were found to be 102.1 cm^2^ C^−1^ at 558 nm, 243.4 cm^2^ C^−1^ at 1390 nm, and 210.6 cm^2^ C^−1^ at 1825 nm. The parameters concerning the switching performance of the polymers were summarized in Table 3, including Δ*T*%, τ, and CE values. The parameters of PMFTQ and PMFEQ [27] are also presented in the table for comparison purposes. Apparently, all four polymer films revealed satisfactory performance among the D–A type polymers reported previously, which make these four novel polymers promising candidates for electrochromic materials. 

## 4. Conclusions

In this study, four D–A type polymers containing the same quinoxaline derivative as the acceptor unit were prepared and characterized. Primary colors including cyan, green, blue, and purple were facilely obtained through the simple modification of the thiophene structures as the donor units. It was particularly remarkable that the polymers with primary colors could be easily obtained by taking commercially available thiophene derivatives as the donor unit, which could boost the commercialization of the electrochromic polymers and reduce the costs for industrial production. 

## Figures and Tables

**Table 1:** The electrochemical, optical, and band gaps of **M1**–**M4** and their polymers, and the band gaps were obtained from either the optical measurement or the theoretical calculations.

Compounds	*E_onset_*, vs. (Ag-wire) (V)	λ*_max_* (nm)/λ*_onset_* (nm)	*E_g_* ^a^ (eV)	HOMO ^b^ (eV)	LUMO ^c^ (eV)	Δ*E* ^d^ (eV)	HOMO ^d^ (eV)	LUMO ^d^ (eV)
**M1**	0.77	322,461/534	2.32	−5.19	−2.87	2.83	−5.06	−2.23
**M2**	0.90	310,407/517	2.39	−5.32	−2.93	2.89	−5.31	−2.42
**M3**	0.97	314,403/511	2.42	−5.39	−2.97	2.94	−5.31	−2.37
**M4**	1.05	313,403/499	2.49	−5.47	−2.98	2.95	−5.39	−2.44
**P1**	−0.11	427,764/839	1.48	−4.31	−2.83	-	-	-
**P2**	−0.17	377,749/915	1.36	−4.25	−2.89	-	-	-
**P3**	0.06	406,679/840	1.49	−4.48	−2.99	-	-	-
**P4**	0.17	362,560/808	1.54	−4.59	−3.05	-	-	-
PMFTQ ^e^	−0.40	-	1.26	-	-	-	-	-
PMFEQ ^e^	−0.18	-	1.22	-	-	-	-	-

^a^ Calculated from the low energy absorption edges (λ*_onset_*), *E_g_* = 1241/λ*_onset_*; ^b^ HOMO = −*e*(*E_onset_* + 0.02 + 4.4); ^c^ LUMO = HOMO + *E_g_*; ^d^ Calculated by employing the Gaussian 05 program; ^e^ See the reference [27]. LUMO refers to the lowest unoccupied molecular orbital.

**Table 2:** The values of the colorimetry coordinates of **P1** to **P4** under different biased potentials.

Polymer	*E*, vs. (Ag-wire) (V)	CIELAB 1976 (L*a*b*)			CIE 1931 Color coordinates
		L*	a*	b*	
**P1**	−0.8	78.76	−30.32	17.42	x = 0. 30; y = 0.40
	0	82.58	−14.93	10.26	x = 0. 31; y = 0.36
	1.1	87.35	−3.17	5.97	x = 0.32; y = 0.34
**P2**	−0.5	61.81	−33.03	21.24	x = 0.30; y = 0.43
	0.5	67.30	−12.22	15.04	x = 0.32; y = 0.38
	1.05	68.26	−5.88	11.69	x = 0.33; y = 0.36
**P3**	−0.5	64.92	−19.46	−7.40	x = 0.26; y = 0.33
	0.5	71.36	−12.22	3.10	x = 0.30; y = 0.35
	1.1	75.89	−7.69	6.92	x = 0.31; y = 0.36
**P4**	−0.5	46.88	2.26	−20.01	x = 0.26; y = 0.27
	0.75	64.30	−6.79	5.38	x = 0.31; y = 0.35
	1.2	68.60	−3.17	7.96	x = 0.33; y = 0.35

**Table 3:** The optical switching properties of the four polymers.

Compounds	λ (nm)	Optical contrast (Δ*T*%)	Response time (s)	Coloration efficiency (CE, cm^2^ C^−1^)
**P1**	760	47	0.24	264.7
	1420	65	1.21	247.6
	1560	70	0.60	300.0
**P2**	750	38	1.00	185.4
	1000	54	0.80	135.3
	1900	70	1.80	254.4
**P3**	410	25	2.32	171.3
	690	34	3.00	252.8
	1560	80	0.83	350.0
**P4**	558	23	0.34	102.1
	1390	60	0.83	243.4
	1825	77	0.84	210.6
PMFTQ ^a^	560	18	1.6	-
	790	60	1.4	-
	1300	80	1.8	-
PMFEQ ^a^	443	22	0.8	-
	780	28	0.7	-
	1600	76	1.0	-

^a^ See the reference [27].

## Supplementary Materials

The following data are available online at www.mdpi.com/2073-4360/9/12/656/s1. Figure S1: ^1^H NMR and ^13^C NMR of Compound 4; Figure S2: ^1^H NMR and ^13^C NMR of **M1**; Figure S3: ^1^H NMR and ^13^C NMR of **M2**; Figure S4: ^1^H NMR and ^13^C NMR of **M3**; Figure S5: ^1^H NMR and ^13^C NMR of **M4**; Figure S6: The redox stabilities of the polymers in the electrolyte; Figure S7: The electrochromic stabilities of **P2**; Figure S8: The electrochromic stabilities of **P3**; Figure S9: The electrochromic stabilities of **P4**.
