# Supplementary material for: The Availability of Neutral Cyan, Green, Blue and Purple Colors from Simple D–A Type Polymers with Commercially Available Thiophene Derivatives as the Donor Units
Source: Polymers (Basel). 2017 Nov 29;9(12):656. doi: 10.3390/polym9120656 (PMC6418635; doi:10.3390/polym9120656)
Supplement: Supplementary file 1 [file polymers-09-00656-s001.pdf]

Supporting Information

The Availability of Neutral Cyan, Green, Blue and Purple Colors from Simple D–A Type Polymers with Commercially Available Thiophene Derivatives as the Donor Units

Lingqian Kong [1,2], Min Wang [3], Xiuping Ju [1], Jinsheng Zhao [2,*], Yan Zhang [2] and Yu Xie [4,*]

[1] Dongchang College, Liaocheng University, Liaocheng 252059, China; lingqiankong@126.com (L.K.); jxp1127@163.com (X.J.)

[2] Department of Chemistry, Liaocheng University, Liaocheng 252059, China; zhang_yan1219@126.com

[3] Liaocheng People's Hospital, Liaocheng 252000, China; wangmin1724@163.com

[4] College of Environment and Chemical Engineering, Nanchang Hangkong University, Nanchang 330063, China

[*] Correspondence: j.s.zhao@163.com or zhaojinsheng@lcu.edu.cn (J.Z.); xieyu_121@163.com (Y.X.); Tel.: +86-635-853-9607 (J.Z.)

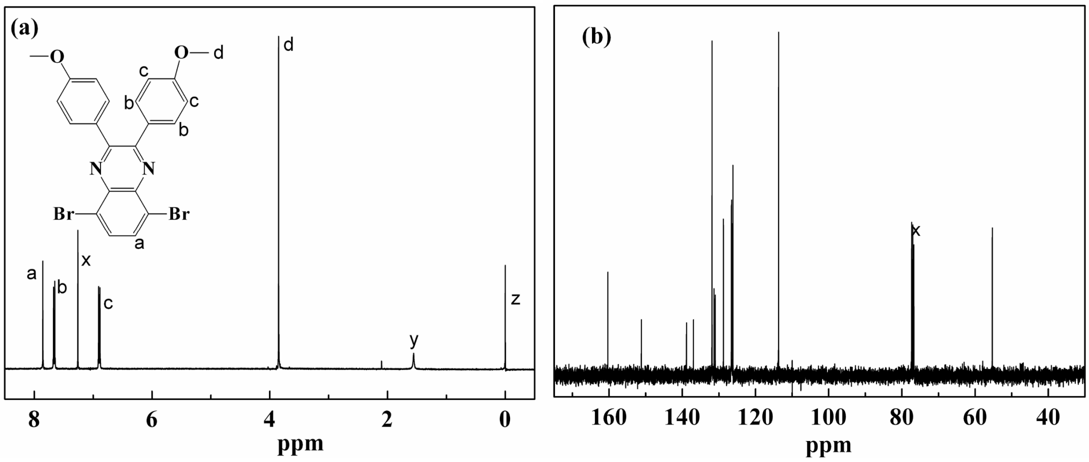

Fig. S1 (a) ^{1}H NMR spectrum of Compound 4. Solvent peak at δ = 7.26 ppm, water speak at δ = 1.56 ppm and tetramethylsilane peak at δ=0 ppm were marked by 'x', 'y' and 'z', respectively. (b) ^{13}C NMR spectrum of 5,8-dibromo-2,3-bis(4-methoxyphenyl)quinoxaline in $CDCl_3$. Solvent peak at δ = 77.3 ppm was marked by 'x'.

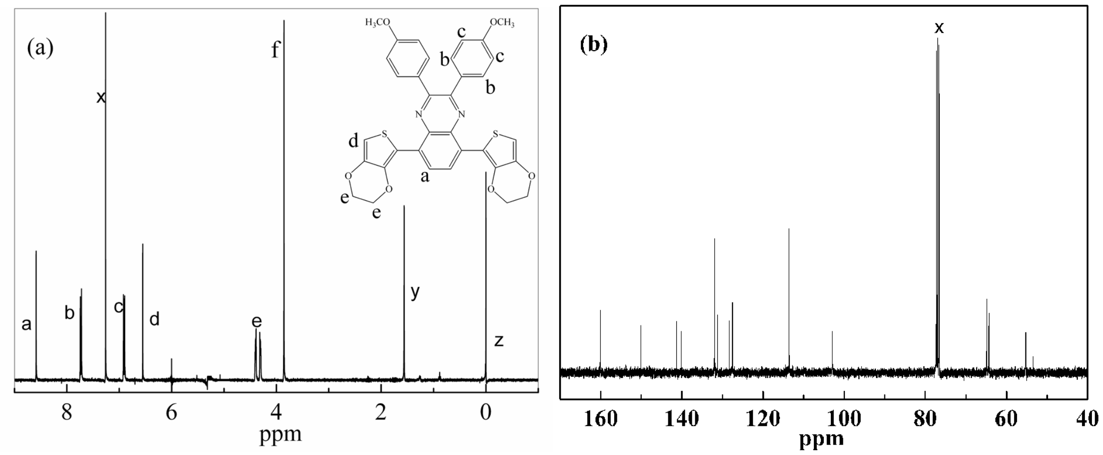

Fig. S2 (a) ^{1}H NMR spectrum of M1 in $CDCl_3$. Solvent peak at δ = 7.26 ppm, water speak at δ = 1.56 ppm and tetramethylsilane peak at δ = 0 ppm were marked by 'x', 'y' and 'z', respectively. (b) ^{13}C NMR spectrum of M1 in $CDCl_3$. Solvent peak at δ = 77.3 ppm was marked by 'x'.

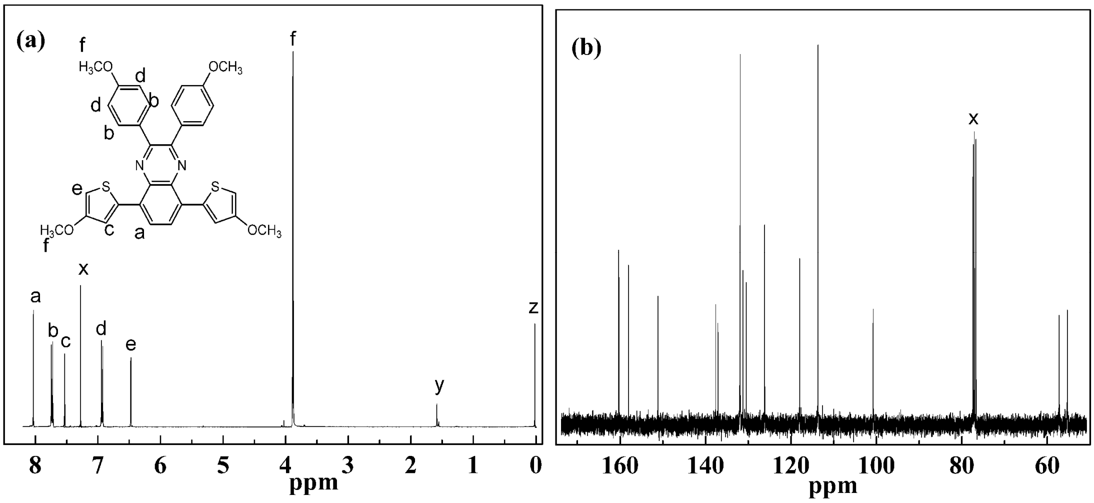

Fig. S3 (a) ^{1}H NMR spectrum of M2 in $CDCl_3$. Solvent peak at δ = 7.26 ppm, water speak at δ = 1.56 ppm and tetramethylsilane peak at δ = 0 ppm were marked by 'x', 'y' and 'z' respectively. (b) ^{13}C NMR spectrum of M2 in $CDCl_3$. Solvent peak at δ = 77.3 ppm was marked by 'x'.

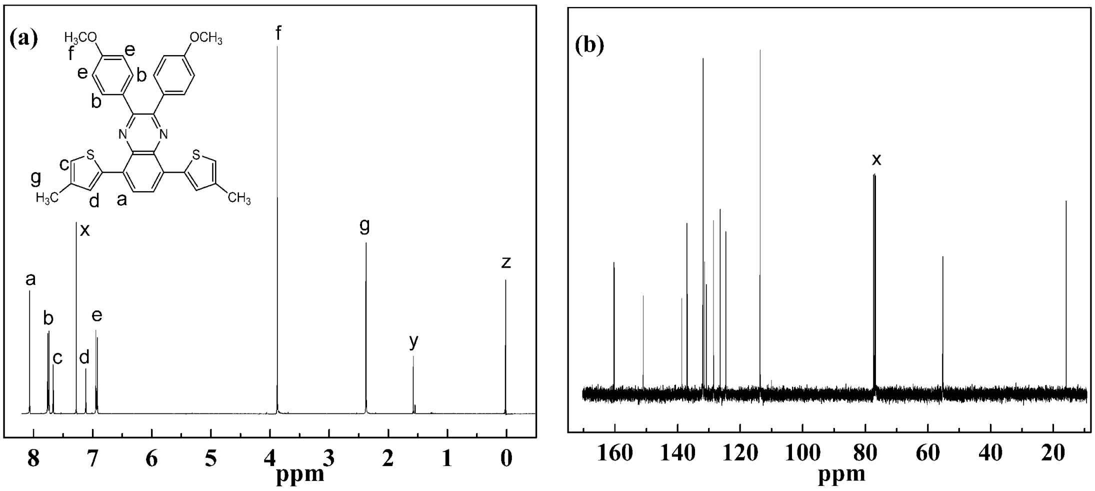

Fig. S4 (a) ^{1}H NMR spectrum of M3 in $CDCl_3$. Solvent peak at δ = 7.26 ppm, water peak at δ = 1.56 ppm and tetramethylsilane peak at δ = 0 ppm were marked by 'x', 'y' and 'z' respectively. (b) ^{13}C NMR spectrum of M3 in $CDCl_3$. Solvent peak at δ = 77.3 ppm was marked by 'x'.

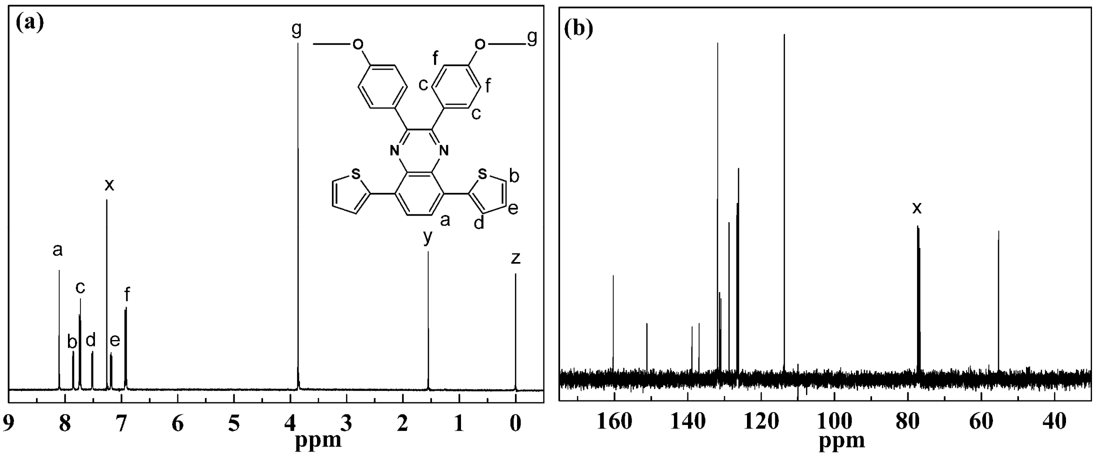

Fig. S5 (a) ^{1}H NMR spectrum of 2,3-bis(4-methoxyphenyl)-5,8-di(thiophen-2-yl)quinoxaline (M4) in $CDCl_3$. Solvent peak at δ = 7.26 ppm, water peak at δ = 1.56 ppm and tetramethylsilane peak at δ = 0 ppm were marked by 'x', 'y' and 'z', respectively. (b) ^{13}C NMR spectrum of 2,3-bis(4-methoxyphenyl)-5,8-di(thiophen-2-yl)quinoxaline (M4) in $CDCl_3$. Solvent peak at δ = 77.3 ppm was marked by 'x'.

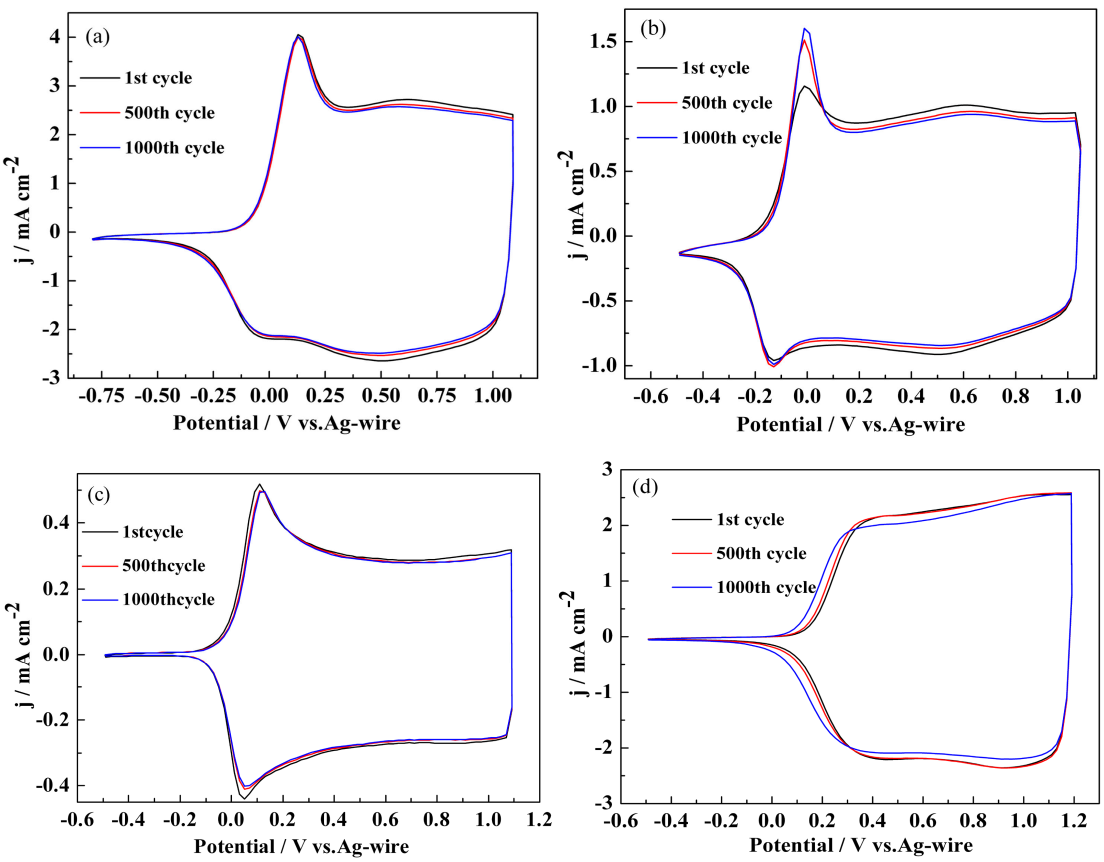

Fig. S6 Electrochemical stability of the P1 (a), P2 (b), P3 (c) and P3 (d) films in the monomer-free 0.2 M $TBAPF_6$–ACN–DCM solution at a scan rate of 200 mV s^{-1}.

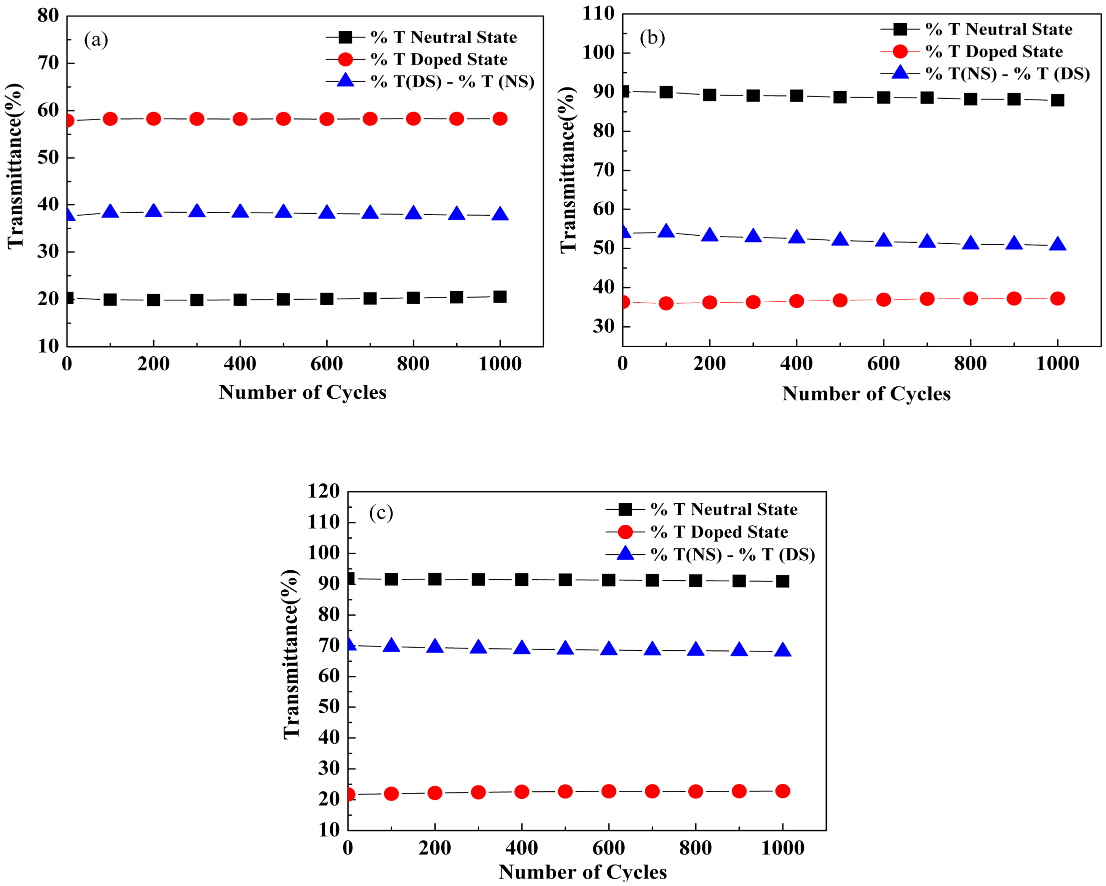

Fig. S7. Electrochromic stabilities of P2 at 750 nm (a), 1000 nm (b), and 1900 nm (c) in the electrolyte.

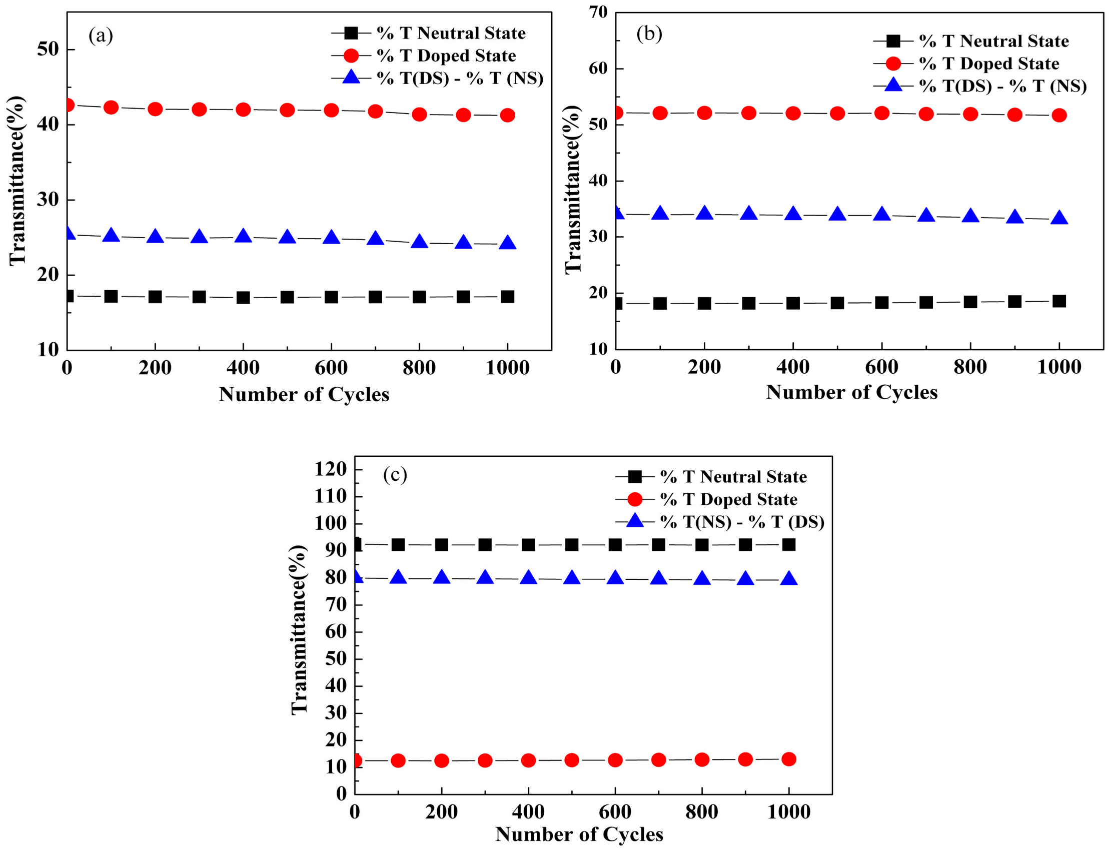

Fig. S8. Electrochromic stabilities of P3 at 410 nm (a), 690 nm (b), and 1560 nm (c) in the electrolyte.

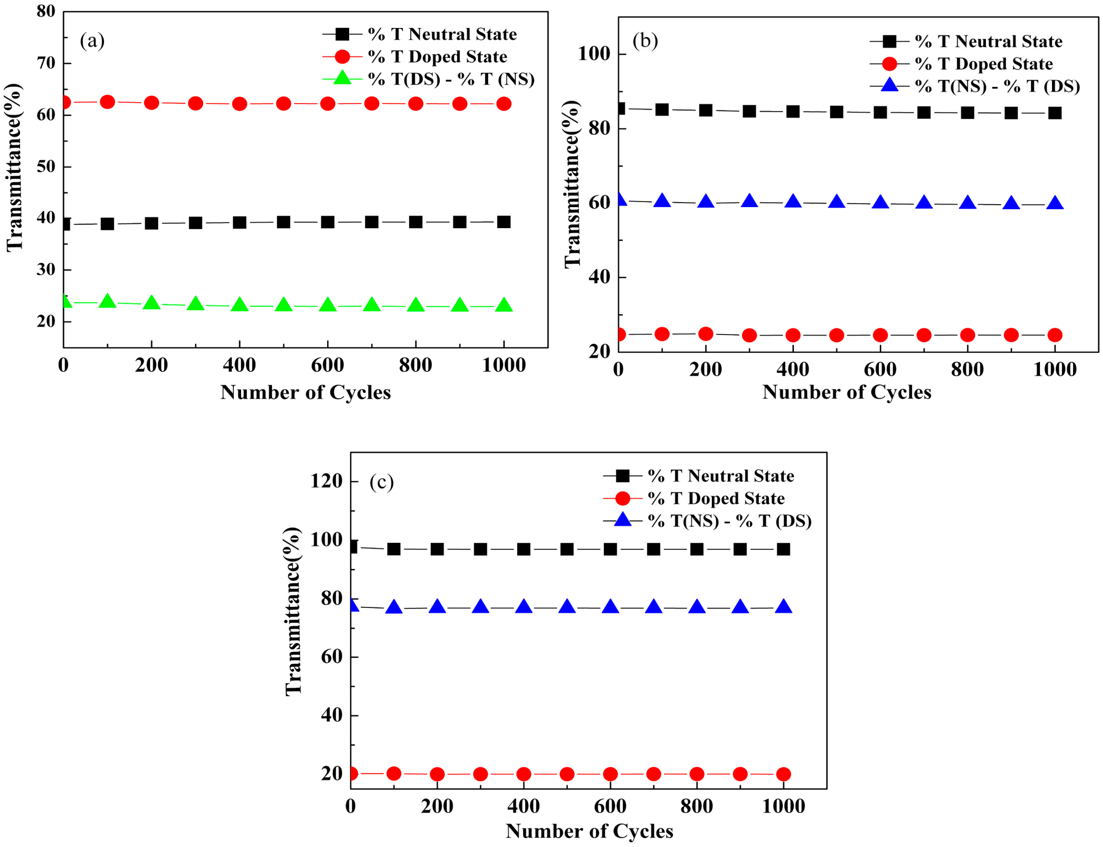

Fig. S9. Electrochromic stabilities of P4 at 680 nm (a), 1115 nm (b), and 1490 nm (c) in the electrolyte.
